# Leveraging machine learning in nursing: innovations, challenges, and ethical insights

**DOI:** 10.3389/fdgth.2025.1514133

**Published:** 2025-05-23

**Authors:** Sophie So Wan Yip, Sheng Ning, Niki Yan Ki Wong, Jeffrey Chan, Kei Shing Ng, Bernadette Oi Ting Kwok, Robert L. Anders, Simon Ching Lam

**Affiliations:** ^1^ School of Nursing and Health Studies, Hong Kong Metropolitan University, Hong Kong, Hong Kong SAR, China; ^2^ School of Computer Science, University of Leeds, Leeds, United Kingdom; ^3^ School of Public Health, Li Ka Shing Faculty of Medicine, The University of Hong Kong, Hong Kong, Hong Kong SAR, China; ^4^ Department of Mathematics, Faculty of Natural Sciences, Imperial College, London, United Kingdom; ^5^ Department of Diagnostic Radiology, School of Clinical Medicine, The University of Hong Kong, Hong Kong, Hong Kong SAR, China; ^6^ Department of Ocean Science, Hong Kong University of Science and Technology, Hong Kong, Hong Kong SAR, China; ^7^ School of Nursing, University of Texas at El Paso, El Paso, TX, United States; ^8^ School of Nursing, Tung Wah College, Hong Kong, Hong Kong SAR, China

**Keywords:** machine learning, artificial intelligence, digital health, predictive analytics, ethical considerations, interdisciplinary collaboration

## Abstract

**Aim/objective:**

This review aims to provide a comprehensive analysis of the integration of machine learning (ML) (1) in nursing by exploring its implications on patient care, nursing practices, and healthcare delivery. It highlights current applications, challenges, ethical considerations, and the potential future developments of ML in nursing.

**Background:**

With the advent of ML in healthcare, the nursing profession stands on the cusp of a transformative era. Despite the technological advancements, discussions on the utilization of ML in nursing, which are crucial for advancing the profession, are lacking. This review seeks to fill this gap by examining the balance between technological innovation and the human-centric nature of nursing.

**Design:**

This narrative review employs a detailed search strategy across several databases, including PubMed, Embase, MEDLINE, Scopus, and Web of Science. It focuses on articles that were published from January 2019 to December 2023. Moreover, this review aims to illustrate the current use, challenges, and future potential of ML applications in nursing.

**Methods:**

Inclusion criteria targeted articles that focus on ML application in nursing, challenges, ethical considerations, and future directions. Exclusion criteria omitted opinion pieces and nonrelevant studies. Articles were categorized into themes, such as patient care, nursing education, operational efficiency, ethical considerations, and future potential, thus facilitating a structured analysis.

**Results:**

Findings demonstrate that ML has significantly enhanced patient monitoring, predictive analytics, and preventive care. For example, the COMPOSER deep learning model for early sepsis prediction was associated with a 1.9% absolute reduction (17% relative decrease) in in-hospital sepsis mortality and a 5.0% absolute increase (10% relative increase) in sepsis bundle compliance. In nursing education, ML has improved simulation-based training by facilitating adaptive learning experiences that support continual skill development. Furthermore, ML contributes to operational efficiency through automated staffing optimization and administrative task automation, thus reducing nurse workload and enhancing patient care outcomes. However, key challenges include ethical considerations, such as data privacy, algorithmic bias, and patient autonomy, which necessitate ongoing research and regulatory oversight.

**Conclusions:**

ML in nursing offers transformative potential across patient care, education, and operational efficiency, which is balanced by significant challenges and ethical considerations. Future directions include expanding clinical and community applications, integrating emerging technologies, and enhancing nursing education. Continuous research, ethical oversight, and interdisciplinary collaboration are essential for harnessing ML's full potential in nursing to ensure that its advancements improve patient outcomes and support nursing professionals without compromising core nursing values.

## 1 Introduction

The advent of machine learning (ML) (1) in healthcare particularly within the nursing profession heralds a transformative era (2–5). Unlike physicians who primarily use ML for diagnostic support, nurses must integrate ML into continuous patient monitoring, bedside care, and administrative workflow management. Nursing workflows involve high-frequency, real-time decision-making, thus requiring adaptive artificial intelligence (AI) tools that assist in patient triage, fall prevention, and workload management. Additionally, nurses face high cognitive and emotional demands because they provide direct patient care and education. As such, ML models that enhance rather than replace human interaction are necessary. These unique challenges emphasize the need for nursing-specific ML applications that prioritize real-time decision support, workload optimization, and patient-centered AI interventions. As a critical component of patient care, the healthcare industry continually evolves and adapts to technological advancements. However, reviews and discussions about utilizing ML in nursing (6), which are important in advancing this technology, are lacking. In this review, the integration of ML in nursing is investigated by exploring its multifaceted implications on patient care, nursing practices, and healthcare delivery. The primary objective is to provide a thorough analysis of how ML applications are currently utilized in nursing, the challenges and ethical considerations they bring, and the potential future developments they promise (7). Therefore, we need to explore the balance between technological innovation and the inherent human-centric nature of nursing by examining how ML can augment patient outcomes while maintaining the compassionate core of nursing (8). This narrative review contributes to the growing discourse on the role of advanced technology in healthcare particularly in enhancing and reshaping the nursing profession in the coming second quarter of the 21st century. Thus, it offers a comprehensive overview of ML's current applications, educational impacts, operational efficiencies, ethical dilemmas, implementation challenges, and future potential in nursing (9).

## 2 Methods

This narrative review comprehensively illustrates the current use of ML applications in nursing, the challenges and ethical considerations, and potential future advancements. A detailed search strategy was employed by utilizing a range of keywords that are associated with ML and its applications within various sectors of healthcare, including “machine learning,” “artificial intelligence,” “nursing,” “healthcare,” “clinical,” “education,” “training,” and “management.” The databases PubMed, Embase, MEDLINE, Scopus, and Web of Science were systematically searched. Given the vast number of publications that are related to ML in nursing, the inclusion of all available studies across all periods was neither feasible nor methodologically appropriate. Instead, a targeted approach was adopted to ensure the inclusion of highly relevant and recent studies that align with current advancements and practical applications in nursing. PubMed, MEDLINE, Embase, Scopus, and Web of Science were selected for their comprehensive coverage of healthcare, clinical, and interdisciplinary AI research. PubMed and MEDLINE focus on nursing and clinical studies. Embase provides pharmaceutical and European research insights. Scopus offers broad scientific coverage, while Web of Science enables citation tracking to identify influential works. Studies were prioritized based on recency, impact, and direct applicability to nursing practice to maintain relevance and methodological rigor. Meanwhile, older foundational papers were included selectively if they provided critical background knowledge. This approach balances breadth and depth, thus ensuring that the review remains focused, up to date, and practically relevant to nursing professionals and researchers. The scope of the search was narrowed to English full-text articles that were published between January 2019 and December 2024.

Inclusion criteria were articles that specifically focused on the application of ML in nursing, tackled challenges or ethical considerations related to its use, or explored future directions in the field. Exclusion criteria were set to omit opinion pieces, editorials, and conference abstracts, as well as studies that did not directly relate to ML applications in nursing contexts.

Following the search, articles were categorized based on their primary focus areas: patient care, nursing education, operational efficiency, ethical considerations, and potential of ML in nursing. These categories were developed inductively during an initial scoping of the literature and refined through team discussions to reflect recurring themes across the included studies. We maintained the general term “ML” in the descriptions to preserve the integrity of the original reports when the studies did not specify the type of ML model or tool used. Although these works are not based on a single published framework, they align with the core domains where ML integration is most impactful in nursing. This categorization facilitated a structured analysis by focusing on identifying common themes, challenges, and ethical considerations associated with the application of ML. The review process culminated in synthesizing these findings to discuss future directions and the potential use of ML in nursing.

Additionally, the current review was conducted in compliance with the Scale for the Assessment of Narrative Review Articles quality checklist (10) to ensure a high standard of narrative review integrity and thoroughness. While a narrative review allows for a comprehensive synthesis of the literature, it inherently involves selection bias because of the need for curated inclusion criteria. Unlike systematic reviews, which aim for exhaustive data collection, this review focused on identifying key trends, practical applications, and major challenges in ML for nursing rather than compiling all available studies. This approach was necessary to distill meaningful insights for nursing practitioners, educators, and policymakers. Clear inclusion and exclusion criteria were applied to mitigate potential biases. This application ensured that studies with high clinical relevance, methodological rigor, and practical significance were prioritized. Future research can expand upon this review by conducting systematic reviews or meta-analyses that quantitatively assess ML's effectiveness in specific nursing applications. No ethical approval was needed for a review study.

## 3 Current applications of ML in clinical nursing

### 3.1 Patient monitoring, predictive analytics, and preventive care

ML in patient monitoring represents a substantial leap from traditional methods. With the advent of advanced algorithms, ML can analyze vast amounts of patient data in real time, thereby enabling the early detection of potential health issues. For example, the deployment of the COMPOSER deep learning model for early sepsis prediction was associated with a 1.9% absolute reduction (17% relative decrease) in in-hospital sepsis mortality and a 5.0% absolute increase (10% relative increase) in sepsis bundle compliance (11). Similarly, Huang and colleagues conducted a comprehensive review of the application of ML-powered predictive analytics in hospital readmission, including logistic regression, random forests, and ensemble learning approaches (12). Their findings indicate that ML models significantly enhance the early identification of high-risk patients in their hospital stay, thus enabling timely interventions and reducing overall readmission rates. ML algorithms can process data from various sources, such as vital signs, medical history, and real-time monitoring devices, to predict patient deterioration (13). For example, systems such as the Rothman Index use ML to provide a single, continuously updated score that reflects the patients’ condition, thereby helping nurses identify patients who are at risk of sudden decline (14). ML models are increasingly used in managing chronic diseases, such as diabetes and heart failure. These systems can predict exacerbations by analyzing trends in patient data, thereby allowing for timely interventions. These interventions can enhance patient outcomes and reduce hospital readmissions.

Preventive care is another area where ML makes a significant impact. ML analyzes patient data and identifies risk factors to help nurses devise preventive strategies that they can implement (15). ML models can identify patients who are at high risk for certain conditions, such as hospital-acquired infections or falls, thus allowing for preemptive measures. In the broad scope of public health, ML aids in identifying patterns and trends in population data. Thus, it can guide nurses in community health initiatives and resource allocation.

### 3.2 Personalized patient care

ML revolutionizes personalized care, which is a cornerstone in the nursing profession. ML enables nurses to provide tailored care by leveraging patient information. Moreover, it can help develop individualized care plans by using historical patient data. For instance, in cancer treatment, ML algorithms analyze patient responses to different treatments; thus, these algorithms can aid in crafting personalized therapy regimens (16). Tools powered by ML can also offer personalized health recommendations.

### 3.3 Clinical decision support systems

Clinical decision support systems (CDSSs) with ML capabilities transform how nurses interact with clinical information, thus improving decision-making processes in patient care. ML algorithms assist in diagnosing complex conditions by analyzing patient data against vast medical databases. Typically, deep learning models or supervised learning techniques are employed in these CDSSs to enhance diagnostic accuracy and personalized recommendations. For instance, ML can help interpret diagnostic images or identify subtle patterns in patient symptoms that may indicate a specific diagnosis. ML-driven CDSSs can suggest optimal treatment plans based on individual patient profiles. These systems can recommend personalized treatment options that may yield favorable outcomes by considering a patient's medical history, current health status, and genetic information (17). ML tools are also used in medication management. For instance, they help nurses to prevent adverse drug events. These tools can analyze patient records and current medications to flag potential drug interactions or contraindications, thus ensuring patient safety (18).

### 3.4 Integrating wearable technology

In nursing care, the integration of wearable technology, which is enhanced by ML, offers continuous monitoring and data collection outside of traditional healthcare settings. Wearables that are equipped with ML algorithms can monitor vital signs and detect abnormalities; they can alert nurses to potential issues in real time even when the patient is at home (19). The data collected from wearables provide valuable insights into patient health trends, thus enabling proactive management of chronic conditions and lifestyle diseases (20). The integration of ML in nursing opens new avenues in patient care by enhancing the capabilities of nurses in monitoring, personalized care, decision-making, preventive measures, and remote patient management. The roles of nurses further expand as these technologies continue to evolve; this scenario demands a balance between technological proficiency and compassionate care (21).

## 4 Enhancing nursing education and training with ML

### 4.1 Simulation-based training

ML significantly enhances simulation-based training (SBT), which is crucial for preparing nurses for real-world scenarios (22). Advanced ML algorithms can create highly realistic and complex patient scenarios for simulation training. These simulations can mimic various clinical conditions, thus providing nurses with diverse and challenging training environments. Empirical studies highlight the effectiveness of SBT in nursing education (23). Conducted a systematic review that demonstrated that SBT significantly improves knowledge retention, confidence, and satisfaction among nurse trainees. Furthermore, their findings suggest that SBT provides a safe and controlled environment for practicing critical decision-making. This notion aligns with the role of ML-driven simulations in enhancing interactive and adaptive learning experiences. Integrating ML with simulation allows training programs to analyze learner performance in real time and provide personalized feedback, thus further optimizing competency-based learning. This evidence supports the argument that ML-enhanced simulations offer a structured approach to skill development, thereby reinforcing theoretical learning while preparing nurses for dynamic clinical settings.

ML-driven simulations can adapt to the skill level of the nurse by providing customized training that meets individual learning needs. The simulation scenarios can become increasingly complex as a nurse's skills improve, thereby ensuring continuous skill development. Utilizing ML in simulations allows immediate and detailed feedback on the nurse's performance (24). The uses of ML in simulations include assessing clinical decision-making, procedural skills, and adherence to best practices. Thus, a deep understanding and reflection on clinical actions can be developed.

### 4.2 Continual learning and skill development

ML also plays a crucial role in continual professional development and skill enhancement for nurses. ML algorithms can analyze a nurse's educational background, professional experience, and learning style to recommend personalized learning pathways. This tailored approach ensures that nurses receive relevant and engaging educational content. ML can provide just-in-time learning resources, such as relevant guidelines or research findings, at the point of care (25). This immediate access to information supports nurses in making informed decisions in dynamic clinical environments. ML tools can track learning progress over time by identifying areas where a nurse may need additional training or resources. This ongoing assessment ensures that nurses remain competent in their practice and up to date with the latest healthcare advancements.

### 4.3 Collaborative learning environments

ML facilitates collaborative and interactive learning environments, thereby enhancing the educational experience for nursing professionals. ML-powered platforms can connect nurses globally, which fosters collaborative learning (26). These platforms can recommend peer discussions, shared resources, and collaborative projects based on common interests or learning goals. Incorporating gamification elements through ML can increase engagement and motivation (27). For example, virtual clinical challenges or competitions can enhance learning and skill acquisition in a fun and interactive way.

### 4.4 Addressing the digital skill gap

Addressing the digital skills gap in nursing becomes crucial as technology becomes increasingly integral to healthcare. ML can help identify specific gaps in digital skills among nursing staff and recommend appropriate training programs (28). In this way, all nurses can be competent in utilizing digital tools and technologies in their practice. Incorporating ML and data science fundamentals into nursing curricula is essential. This education can empower nurses to understand and use ML tools effectively in their practice.

The application of ML in nursing education and training is transformative. Thus, it offers personalized, adaptive, and interactive learning experiences. Nursing professionals can stay at the forefront of healthcare innovation by embracing these technologies; these advancements ensure that nursing professionals are well equipped to deliver high-quality patient care in a highly digital world (28).

## 5 Operational efficiency and workflow optimization in nursing through ML

### 5.1 Resource management and staffing optimization

ML significantly contributes to the efficient allocation and management of nursing resources. In light of the global shortage of nurses, which the World Health Organization projects to exceed 10 million healthcare workers by 2030, ML-based resource management tools are especially critical (29). These systems can help healthcare institutions mitigate staffing shortfalls by forecasting patient demand, optimizing nurse scheduling, and automating nonclinical tasks. In doing so, ML allows nursing professionals to concentrate on direct patient care and high-order clinical decision-making, thereby maximizing the effectiveness of limited human resources. For example, a study conducted in Hong Kong developed an automatic nurse roster scheduling system based on open-source operational research and optimization algorithms, which aimed to enhance workforce management and improve patient care delivery. This AI-driven approach helps optimize nurse scheduling, thereby improving workforce management and patient care delivery. The implementation demonstrated the potential of ML techniques to enhance the efficiency of nursing staff allocation in healthcare settings (30). Moreover, ML algorithms can predict patient influx and hospital occupancy rates. Thus, they can help in effective nurse staffing. These systems forecast the required staffing levels by analyzing historical data to ensure optimal patient care without overburdening the nursing staff. ML can also assist in creating shift schedules that consider nurse preferences, skill mix, and workload, thus leading to increased job satisfaction and reduced burnout. ML tools can optimize the allocation of resources, such as medical equipment and rooms, which ensures that these resources are used efficiently and are readily available when needed (31).

### 5.2 Administrative task automation

Reducing the administrative workload of nurses through ML enhances their focus on patient care. ML algorithms can facilitate fast and accurate patient documentation by extracting key information from speech or text, thus significantly reducing the time that nurses spend on paperwork (32). ML can identify inefficiencies in various nursing workflows (e.g., medication administration, patient admission, and discharge processes) and suggest improvements, thereby saving time and reducing errors.

### 5.3 Enhanced communication and coordination

ML improves communication and coordination among healthcare teams. These improvements are vital for effective nursing care. ML-driven tools can anticipate the need for communication between nurses and other healthcare professionals, thus prompting timely interactions that are crucial for patient care (33–35). ML tools analyze patient data to facilitate coordinated care planning among different healthcare providers, which ensures that all aspects of patient care are addressed in a unified manner.

### 5.4 Monitoring and improving quality of care

ML plays a pivotal role in monitoring and enhancing the quality of nursing care. ML algorithms can analyze various quality metrics, such as patient outcomes, readmission rates, and patient satisfaction scores; thus, they can provide insights into areas of improvement in nursing care (12, 36–39). ML tools can offer real-time suggestions for improving care quality, such as recommending evidence-based practices and alerting to potential issues before they escalate.

### 5.5 Managing patient flow and hospital logistics

The efficient management of patient flow and hospital logistics is another area where ML significantly contributes (40, 41). ML algorithms can predict peak times for admissions and discharges, thus enabling good planning and smooth patient flow. In this scenario, wait times can be reduced, and patient satisfaction can be improved. ML can optimize the logistics of hospital operations, such as the timely delivery of supplies and the efficient use of medical equipment. Optimizing these hospital operation logistics ensures that necessary resources are always available, thereby reducing delays in patient care.

### 5.6 Predictive maintenance of medical equipment

ML aids in the predictive maintenance of medical equipment, which is crucial for uninterrupted nursing care. On the basis of usage patterns and historical maintenance data, ML algorithms can predict equipment malfunctions before they occur. This preemptive approach minimizes downtime and ensures that nurses have functioning equipment when needed. ML analyzes equipment usage and conditions to help schedule maintenance activities by minimizing disruption to nursing activities and patient care (42).

ML significantly enhances operational efficiency and workflow optimization in nursing. ML automates administrative tasks, optimizes resource management, and improves communication and quality of care, thereby allowing nurses to focus on direct patient care. Additionally, the predictive capabilities of ML in managing patient flow and maintaining medical equipment ensure a smooth and efficient healthcare environment. This transformation benefits nurses in terms of reduced workload and increased job satisfaction. It also leads to improved patient outcomes and overall healthcare efficiency.

## 6 Ethical considerations and patient safety in the integration of ML in nursing

One of the primary ethical concerns in applying ML in nursing is the potential for algorithmic bias. If ML models are trained on data that reflect historical biases or underrepresent certain populations, they may produce discriminatory outcomes. For example, an ML model designed to predict patient risk may underestimate the needs of minority groups if the training data are not representative. These biases can be mitigated by ensuring that ML models are developed using diverse and inclusive datasets and undergo regular audits to identify and correct any disparities. One solution is the implementation of bias-aware ML training protocols, which require AI developers to test models systematically against diverse nursing populations to ensure fair representation (43). Additionally, adopting explainable AI models can increase transparency, thus allowing nurses to understand and challenge ML-generated recommendations if biases are detected (44). Hospitals can also establish nursing-AI ethics committees, which will be responsible for continuously evaluating ML applications; the establishment of these bodies can ensure that ML applications align with patient-centered care values and do not inadvertently reinforce healthcare disparities (45).

### 6.1 Data privacy and security

One of the foremost ethical considerations in the use of ML in nursing is the protection of patient data (46). Implementing robust data privacy measures is crucial to protecting sensitive patient information. ML systems must comply with healthcare regulations, such as the Health Insurance Portability and Accountability Act (HIPAA) to ensure data confidentiality. Nurses must be trained in secure data handling practices. As such, ML systems should incorporate advanced encryption methods and access controls to prevent data breaches. Federated learning techniques, which train ML models across multiple institutions without transferring patient data, can be employed to minimize privacy risks and further enhance data security (47). Additionally, role-based AI governance models ensure that only authorized nursing professionals can access sensitive patient insights that have been derived from ML analytics; this security measure reduces the risk of misuse and enhances compliance with data protection regulations, such as General Data Protection Regulation and Health Insurance Portability and Accountability Act (48).

### 6.2 Addressing bias and fairness in ML models

The issue of bias in ML algorithms is a significant concern because it can lead to unequal care delivery. Ensuring that ML models are trained on diverse datasets is crucial to preventing bias. Nurses and healthcare professionals should be aware of the potential for bias and work toward mitigating the impact of bias on patient care. The continuous monitoring and evaluation of ML models are necessary to ensure fairness and equity in healthcare delivery (49). This approach includes periodic reviews and updates to algorithms as additional data become available.

Transparency and the ability to explain are key ethical considerations in using ML-based decision-support systems in nursing. Nurses must understand how these systems generate recommendations and know their limitations. Black-box models that provide outputs without clear explanations can undermine trust and hinder effective decision-making. This issue can be addressed by developing interpretable ML models and providing nurses with training on how to use and interpret these systems. This solution will enable nurses to make informed decisions and maintain their professional autonomy while benefiting from the insights provided by ML.

### 6.3 Legal and ethical implications

The integration of ML in nursing also brings several legal and ethical implications that must be carefully considered (50). Therefore, clarifying the accountability and responsibility in decision-making supported by ML is crucial (51). Nurses must understand how much they can rely on ML recommendations and where their professional judgment is paramount. Ensuring informed consent is critical particularly when using ML tools in patient care. Patients should be informed about how their data are used and the role of ML in their treatment plans (52).

As the application of ML in nursing continues to evolve, engaging in ongoing ethical reflection and dialogue is crucial. Beyond addressing ethical concerns, ML presents unique opportunities for nursing that extend beyond conventional applications in medicine. For instance, ML-driven mentorship programs can analyze nurses’ past training experiences and recommend personalized career development paths to enhance retention and skill progression. AI-assisted bedside care, such as voice-activated documentation tools and ML-powered decision support systems, can reduce the administrative burden on nurses, thus allowing them to focus on patient interaction. Additionally, digital nursing assistants can provide real-time guidance based on evidence-based protocols to support clinical decision-making in high-pressure environments.

Developing guidelines and best practices for the ethical use of ML in healthcare with a focus on nursing is essential. These guidelines should address several issues, such as data privacy, informed consent, algorithmic fairness, and the role of nurses in developing and implementing ML systems. Proactively addressing these ethical considerations allows the nursing profession to harness the benefits of ML while upholding its commitment to patient well-being and social justice.

### 6.4 Patient autonomy and trust

Maintaining patient autonomy and trust in an increasingly automated healthcare environment is essential for ethical nursing practice (53). Nurses must ensure that ML tools enhance rather than replace the involvement of patients in their care decisions. Patients should be provided with clear, understandable information about how ML influences their care. However, cultural attitudes toward AI-driven healthcare interventions vary significantly by region. In the United States and parts of Europe, ML adoption in nursing has been facilitated by strong digital infrastructure and regulatory frameworks. By contrast, digital implementation in developing world settings is relatively immature. Thus, ML may be less easily embraced by a healthcare workforce with less familiarity with digital tools. In low-resource settings, various challenges, such as limited internet access, lack of standardized electronic health records, and low digital literacy among nurses, hinder widespread implementation (54). Transparency about the capabilities and limitations of ML systems is key to building trust among patients and healthcare professionals. Nurses play a crucial role in communicating this information effectively.

### 6.5 Ethical use of predictive analytics

Predictive analytics in healthcare raises ethical questions regarding the use of patient data and the implications of predictions. Nurses must consider the ethical implications of predictive analytics, such as the potential for overreliance on predictions, which may affect clinical judgment. The confidentiality of predictive insights, particularly those related to sensitive health conditions, must be maintained. Nurses have a duty to protect this information and use it responsibly in patient care (55).

### 6.6 Navigating the intersection of technology and human care

The technology and the human aspects of care must be balanced as the nursing industry gradually incorporates ML. Although ML can enhance efficiency and accuracy, maintaining compassion and empathy in patient interactions is crucial (56). Nurses must ensure that the use of technology does not diminish the human connection in care. Ethical training regarding ML use in healthcare should be an integral part of nursing education (57). This training includes understanding the ethical implications of ML and developing strategies to address them.

The integration of ML in nursing results in various ethical considerations and challenges that are related to data privacy, algorithmic bias, legal and ethical implications, patient autonomy, and the intersection of technology with human care. We systematically address these challenges by adopting a nursing-specific ethical framework that encompasses three core components: (i) bias-mitigation protocols that require regular audits of ML systems using nursing-sensitive indicators to detect and rectify algorithmic biases that affect patient care, (ii) a patient-centered transparency model that emphasizes clear communication strategies that enable nurses to explain ML-driven decisions and preserve patient autonomy and trust, and (iii) nursing data privacy guidelines that integrate advanced data protection measures, such as federated learning and differential privacy, coupled with mandatory digital literacy training for nursing staff. Implementing these tailored guidelines will ensure that ML applications not only comply with ethical standards but also resonate deeply with nursing values and responsibilities.

Navigating these challenges requires a thoughtful approach that prioritizes patient safety and ethical standards. Equipping nurses with knowledge and tools is imperative in addressing these issues to ensure that the benefits of ML are realized without compromising the core values of nursing care (58). As the field continues to evolve, ongoing education, ethical oversight, and a commitment to patient-centered care remain essential in harnessing the full potential of ML in nursing.

Table 1 summarizes the key applications of ML in nursing.

**Table 1:** Summary of key applications of machine learning in nursing.

Domains	Applications	Examples
Current applications of machine learning in clinical nursing	• Real-time patient monitoring and predictive analytics for early detection of health issues	(13, 14)
	• Enhancing preventive care by identifying high-risk patients	(15)
	• Personalized patient care using historical data for individualized care plans	(16)
	• Clinical decision support systems that aid diagnosis and medication management	(18)
	• Integration of wearable technology for continuous monitoring	(19, 20)
Enhancing nursing education and training with machine learning	• Simulation-based training for diverse clinical conditions	(22, 24)
	• Continual learning and skill development through personalized learning pathways	(25)
	• Collaborative learning environments connecting nurses globally	(26, 27)
	• Addressing the digital skills gap in nursing	(28)
Operational efficiency and workflow optimization in nursing management through machine learning	• Resource management and staffing optimization	(31)
	• Automation of administrative tasks for efficient patient care	(32)
	• Improved communication and coordination among healthcare teams	(33–35)
	• Monitoring and improving the quality of care	(12, 36–39)
	• Managing patient flow and hospital logistics	(40, 41)

## 7 Challenges and barriers to the implementation of ML in nursing

### 7.1 Technical barriers and infrastructure requirements

The implementation of ML in nursing faces significant technical challenges that must be addressed. The effective use of ML in healthcare requires robust digital infrastructure, including high-speed Internet, adequate storage capacity, and powerful computing resources (59). Integrating ML tools with existing healthcare systems and electronic health records can be complex and time consuming (60). Ensuring compatibility and seamless data flow is crucial for the effective use of ML.

### 7.2 Resistance to change among nursing professionals

Adopting new technologies often meets resistance primarily because of the comfort with established practices. Cultural factors significantly shape the integration and acceptance of ML in nursing practice. For instance, in Hong Kong, a high degree of digital literacy and a generally positive attitude toward technological innovations among healthcare professionals have facilitated the relatively smooth adoption of ML in clinical settings. However, ML implementation may face substantial barriers in regions with limited technological infrastructure or differing cultural attitudes toward patient-provider interactions, such as communities that value face-to-face communication. Hence, tailoring ML solutions to reflect regional cultural expectations, providing culturally sensitive training programs, and ensuring equitable resource allocation are crucial steps to enhance the acceptance and effectiveness of ML technologies in diverse nursing contexts. Fears and misconceptions about ML replacing human jobs or undermining professional judgment can be observed (61–63). Addressing these concerns through education and demonstration of the supportive role of ML is essential, as explored in the context of the impact of AI on labor and human potential (64). Enhancing digital literacy among nursing staff is crucial. As such, providing training and support can help ease the transition to tech-enhanced healthcare environments.

### 7.3 Interdisciplinary collaboration challenges

Effective implementation of ML in nursing requires collaboration across various disciplines. One approach to fostering interdisciplinary collaboration is the establishment of ML-nursing integration committees, where nurses, data scientists, and healthcare administrators codevelop AI-driven solutions that align with clinical needs (65). Additionally, integrating cross-disciplinary education programs, where nursing students receive basic ML training and data scientists are educated on clinical workflows, can enhance mutual understanding and collaboration. A gap in communication and understanding often exists between tech professionals who develop ML solutions and healthcare professionals who use them (66). Bridging this gap is essential for developing user-friendly and relevant ML tools. Therefore, ensuring that nurses have a voice in the development and implementation of ML tools is vital. Their insights can guide the creation of additional effective and practical ML solutions.

### 7.4 Data quality and accessibility issues

The success of ML in nursing heavily relies on the quality and accessibility of data. ML algorithms require large amounts of high-quality data to be effective (67). Inconsistent, incomplete, or biased data can lead to inaccurate predictions and recommendations. Gaining access to diverse and comprehensive datasets can be challenging because of privacy concerns and data silos within healthcare systems.

### 7.5 Ethical and regulatory hurdles

Navigating the ethical and regulatory landscape is a significant challenge in implementing ML in nursing. Ensuring that ML applications comply with healthcare regulations, such as HIPAA in the United States, is crucial. This approach includes safeguarding patient privacy and ensuring data security (68–71). The ethical implications of using predictive analytics in healthcare, such as potential biases and the impact on patient care decisions, must be carefully considered and addressed.

The implementation of ML in nursing is fraught with challenges that range from technical barriers and resistance to change to interdisciplinary collaboration difficulties, data quality issues, and ethical and regulatory hurdles. Addressing these challenges requires a multifaceted approach that involves technological advancements, educational initiatives, collaborative efforts, and regulatory compliance. However, many ML approaches operate on aggregate data. Their outputs tend to obscure individual-level information. Therefore, the possibility of data leakage through ML systems is not necessarily greater than that posed by early digital technologies used in healthcare environments. Innovative strategies to accelerate ML integration in nursing can include the development of nurse-led ML pilot projects, where frontline nursing staff collaborate directly with data scientists to tailor predictive algorithms to real-world clinical workflows. Additionally, establishing hybrid roles, such as clinical informatics nurses, who specialize in patient care and ML technology management, can bridge the existing gap between technological development and practical nursing applications. Furthermore, developing a standardized nursing-centric ML validation framework, which emphasizes explainability and usability from a nursing perspective, will ensure that algorithms align closely with clinical needs and ethical standards. Overcoming these barriers is essential for realizing the full potential of ML in enhancing nursing practices and patient care.

## 8 Future directions and potential of ML in nursing

As we navigate the unfolding landscape of ML (1) in nursing, the horizon is vibrant with potential yet marked by areas that necessitate deep inquiry and collaboration. This journey into the future invites a confluence of rigorous research, innovative technologies, and cross-disciplinary synergy to harness fully the transformative power of ML in nursing. Future research should focus on developing ML algorithms that are specifically tailored to nursing tasks. Unlike ML applications in radiology or diagnostics, nursing workflows involve continuous, real-time decision-making that requires adaptive AI tools. One promising direction is the integration of ML into shift management, where predictive analytics can anticipate patient acuity and recommend optimal nurse–patient assignments. Additionally, ML-enhanced clinical training simulations can help nurses refine skills by providing adaptive, scenario-based learning that is tailored to their competency level. These innovations will not only improve efficiency but also enhance patient safety and nursing job satisfaction.

### 8.1 Expanding applications in clinical and community settings

As ML continues to evolve, its applications in nursing are expected to broaden, which will affect the clinical and community healthcare settings (72). Future developments in predictive analytics are anticipated to offer patient care predictions that are not only precise but also highly personalized. As such, these advancements can enhance preventive care and chronic disease management. Furthermore, ML can be utilized in community health monitoring to identify and address public health issues effectively, track disease outbreaks, and implement targeted health interventions (73).

Before delving into the specific areas of expansion for ML in nursing, the overarching directions of this field must be considered.

Further research is essential to grasp the effectiveness of ML interventions in the nursing practice. Despite the demonstrated potential benefits, such as improved patient outcomes and increased efficiency, the actual impact of these interventions requires rigorous, real-world evaluation. Developing ML algorithms that are tailored to nursing, which can include qualitative data analysis or address unique patient care challenges, represents a significant opportunity for advancement. Moreover, exploring ML's application across various nursing specialties, such as mental health or pediatrics, can offer invaluable insights and lead to meaningful innovations.

The advent of emerging technologies, such as explainable AI and federated learning, stands to reshape the future of ML in nursing significantly. Explainable AI aims to make ML models’ decisions transparent and comprehensible by addressing the opaque nature of some existing systems. This clarity can improve ML's transparency and trustworthiness within nursing, thus encouraging broad clinical adoption. Federated learning offers a way to train robust and representative ML models on decentralized data without compromising patient privacy, which points toward the ethical and effective usage of ML in nursing as these technologies evolve.

Interdisciplinary collaboration will play a pivotal role in navigating the future of ML in healthcare. As primary patient care providers and the largest healthcare professional group, nurses must be at the forefront of developing and implementing ML solutions. Their unique expertise and patient-centered perspective are invaluable. Therefore, ML technologies can enhance rather than undermine the quality and humanity of patient care. Nurses should advocate for patient needs and the nursing profession, as well as work alongside healthcare professionals, data scientists, and ML experts to promote an ethical and effective application of ML that improves patient outcomes and supports nursing practice.

The recent advances in large language models (LLMs), including ChatGPT (74) and similar generative AI tools (75), present new opportunities and challenges for nursing. In clinical practice, LLMs can support nurses by summarizing patient records, assisting in documentation, generating shift reports, and providing instant access to clinical guidelines. In nursing education, LLMs offer personalized tutoring, simulation-based dialogue, and assistance with reflective writing. However, these tools also raise concerns about data privacy, hallucinations (i.e., incorrect or fabricated content), and overreliance on nontransparent outputs. The accuracy, safety, and ethical implications of integrating LLMs into nursing workflows must be evaluated. As this technology continues to evolve, interdisciplinary collaboration and robust governance frameworks will be essential to ensure that LLM applications support nursing values, enhance professional judgment, and maintain the integrity of patient care.

While the current landscape shows great promise in integrating ML into nursing, the existing literature has a noticeable scarcity of experimental or quasi-experimental trials that rigorously assess the impact of these technologies on clinical outcomes, workflow efficiency, and educational effectiveness. Future research can advance the science by prioritizing well-designed pilot studies, randomized controlled trials, and implementation studies that evaluate the safety, efficacy, and cost-effectiveness of ML applications in real-world nursing settings. Such research is essential to move beyond conceptual potential and provide a solid evidence base that informs policy, clinical guidelines, and nursing education reforms. Establishing standardized outcome measures and nursing-specific evaluation frameworks will also be critical to ensure the relevance and transferability of findings across diverse healthcare contexts.

As we venture into this exciting future, these considerations set the stage for a deep exploration into how ML will continue to evolve within nursing.

### 8.2 Integration with emerging technologies

The integration of ML with other emerging technologies is poised to transform nursing practices significantly. The integration of ML with the Internet of Things (76) and wearable technology will allow nurses to gain the capability for continuous, real-time health monitoring. Thus, this advancement offers valuable insights into patient health outside traditional clinical environments. Moreover, the synergy of AI, ML, and robotics is expected in the development of nursing-assistive robots, which will not only aid in patient care but also substantially reduce the physical burden on nurses (77).

### 8.3 Personalized and precision medicine

ML is poised to play a pivotal role in advancing personalized and precision medicine in nursing care. Through ML algorithms, several advancements will occur in the development of highly personalized treatment plans that are tailored to individual patient genetics, lifestyle, and environmental factors; thus, the effectiveness of treatments will be enhanced significantly (78–80). The integration of genomics with ML is anticipated to lead to breakthroughs in understanding disease mechanisms and in the development of new treatment modalities.

### 8.4 Enhancing nursing education with AI and ML

The future of nursing education is set to be significantly reshaped by advancements in AI and ML. According to Glauberman et al., the incorporation of AI-driven platforms will transform nursing education by providing interactive and adaptive learning experiences, simulating a range of clinical scenarios, and tailoring content to meet individual learning styles (81). Furthermore, AI and ML will play a crucial role in supporting lifelong learning for nurses, thus ensuring that they have access to the most current information and training on the latest healthcare technologies and practices.

### 8.5 Ethical AI in nursing

The development of ethical AI is imperative as ML becomes increasingly integrated into nursing (82). Clear ethical guidelines and standards for the use of AI and ML in healthcare must be established to guarantee that these technologies are used responsibly and primarily for the benefit of patients. Future advancements are expected to focus on creating transparent and explainable AI systems, which will allow nurses and patients to understand and trust the decisions made by these technologies.

The future of ML in nursing holds immense potential, thus indicating the transformation in patient care, healthcare operations, and nursing education. Integrating ML with other cutting-edge technologies will likely lead to significant advancements in personalized and precision medicine, enhance the efficiency and effectiveness of nursing practices, and revolutionize how nurses are trained and continue their professional development. However, navigating the ethical implications and maintaining a patient-centered focus becomes important as the technologies evolve. The nursing profession can fully harness the potential of ML to improve patient outcomes and healthcare delivery by balancing technological innovation with compassionate care. The exploration of these future directions not only highlights the exciting possibilities but also underscores the need for ongoing research, collaboration, and ethical consideration in integrating ML within the field of nursing.

As we venture into this future, continuous research should be done to validate the effectiveness of ML applications in nursing, address potential ethical concerns proactively, and ensure that technology enhances rather than replaces the human touch in patient care. The collaboration among nurses, technologists, and other healthcare professionals will be vital in developing solutions that are not only innovative but also grounded in the realities of patient care. Furthermore, the active participation of nurses in shaping the development and application of ML technologies will ensure that these advancements truly reflect the needs and values of patients and healthcare providers.

### 8.6 Limitations

This narrative review has several limitations. First, the search strategy was not exhaustive. Although we aimed to capture a broad representation of ML applications in nursing, the inclusion of articles was not based on a systematic review protocol. As such publication bias or omission of relevant studies may have occurred. Second, the categorization of included studies into thematic domains was conducted inductively and may be subject to interpretation bias. Third, many of the articles cited describe theoretical applications or early-stage developments rather than empirically validated interventions. Thus, some proposed potentials of ML remain speculative. Fourth, given the rapidly evolving nature of AI and ML technologies, the literature may quickly become outdated. Emerging developments, such as large language models, may not yet be fully captured in peer-reviewed research. Finally, while we attempted to include global perspectives, the literature predominantly reflects research from high-resource settings, thus limiting the generalizability of findings to low- and middle-income countries.

These limitations should be considered when interpreting the findings. Future work may benefit from systematic reviews or meta-analyses that are focused on specific ML applications, as well as primary research that tests ML integration in diverse nursing contexts.

## 9 Summary and conclusion

As we conclude this comprehensive exploration of ML in nursing, we can see that ML is not only a fleeting technological trend but also a fundamental component that shapes the future of healthcare. This review has traversed various dimensions of ML's integration into nursing from enhancing patient care and optimizing operational workflows to transforming nursing education and raising significant ethical considerations.

The journey through the current applications of ML in nursing has revealed its immense potential in patient monitoring, personalized care, and decision support systems. These applications underscore ML's capability to augment the efficiency of nursing practices and elevate the quality of patient care. The insights that have been gained in this study point toward a future where nurses, who are aided by advanced algorithms, can deliver highly precise, predictive, and personalized healthcare services. In nursing education, ML emerges as a powerful tool because it offers dynamic, adaptive, and personalized learning experiences. It prepares nursing professionals for a highly digital and data-driven healthcare landscape, thereby ensuring that they remain at the forefront of medical innovation and patient care. Operational efficiency, which is a key concern in healthcare, is notably enhanced through ML, with improved resource management, administrative task automation, and streamlined workflows. These advancements contribute to a highly efficient healthcare system, thus allowing nurses to focus on their primary role of patient care.

However, the integration of ML in nursing is not without challenges. This review has highlighted significant barriers, including technical hurdles, resistance to change, and the need for interdisciplinary collaboration. Addressing these challenges is essential for the successful implementation and acceptance of ML in nursing. Specific actionable strategies include the implementation of comprehensive digital literacy training programs within nursing curricula and ongoing professional development, thereby enabling nurses to utilize ML technologies confidently. Additionally, establishing structured interdisciplinary collaboration frameworks, such as regular workshops or joint taskforces comprising nurses, healthcare administrators, ethicists, and AI experts, can bridge communication gaps and enhance practical ML adoption. Policy-level recommendations involve creating institutional ethical oversight committees, which will be responsible for routinely evaluating ML deployments from ethical, clinical, and patient-safety perspectives. Such solutions can ensure compliance and continuous improvement in nursing practice. Ethical considerations, particularly concerning data privacy, algorithmic bias, and patient autonomy, remain paramount. As the nursing industry continues to incorporate additional ML tools, maintaining ethical standards and prioritizing patient safety and trust are crucial. Developing clear guidelines and continuous ethical education is imperative in navigating these complexities.

Looking ahead, the future directions of ML in nursing are promising and diverse. From expanding applications in clinical and community settings to integration with emerging technologies, such as IoT and genomics, ML is set to revolutionize nursing practices further. However, as we embrace these advancements, we must not lose sight of the core values of nursing: compassion, empathy, and patient-centered care. The harmonious integration of ML within the nursing industry requires a balanced approach that leverages technological capabilities while upholding the human touch that is intrinsic to the profession.

In conclusion, the integration of ML into the nursing field presents a landscape that is rich in opportunities and challenges. As we step into this future, the nursing community, healthcare institutions, and technology developers must collaborate to ensure that ML is used responsibly, ethically, and effectively in enhancing patient care and supporting nursing professionals in their invaluable roles in healthcare. Healthcare administrators should prioritize the development of standardized ML protocols and frameworks that align with ethical and patient safety regulations (83). Nursing educators must integrate AI and ML literacy into training programs, thus ensuring that nurses gain practical experience with these technologies before entering the workforce (84). Implementing these targeted actions can ensure that ML-driven innovations support and enhance nursing practices without compromising the fundamental values of patient-centered care.
